# Lower genital tract infections during pregnancy and adverse pregnancy outcomes: a hospital based observational cohort study

**DOI:** 10.1186/1471-2334-14-S3-E35

**Published:** 2014-05-27

**Authors:** Chaitanya Tellapragada, KE Vandana, Parvati V Bhat, Chythra Rao, Asha Kamath, Satheesha Nayak, V Shashidhar, Shashidhar Acharya, Chiranjay Mukhopadhyay

**Affiliations:** 1 Department of Microbiology and Community Medicine, Kasturba Medical College, Manipal University, Manipal, India; 2 Department of Obstetrics and Gynecology, Melaka Manipal Medical College, Manipal University, Manipal, India; 3 Department of Community Dentistry, Manipal College of Dental Sciences, Manipal University, Manipal, India

## Background

Maternal lower genital tract during pregnancy is a complex niche of microbes that normally inhabit or cause infections in few instances. Association of various microbial flora and adverse pregnancy outcomes is being increasingly explored. The study was aimed to determine the prevalence of lower genital tract infections (LGTI) among pregnant women and to determine the common etiologies of LGTI and their association with adverse pregnancy outcomes, Pre term birth (PTB) and Low birth weight (LBW).

## Methods

A hospital based observational cohort study comprising 486 asymptomatic, antenatal women in the age group of 18-35 and the gestational period of 8-24 weeks was carried out. High vaginal and endocervical swabs were tested for Nugent’s score, culture and PCR for Mycoplasmas and Chlamydia. All the women were followed until delivery.

## Results

Prevalence of LGTI s among study population was 134(28%) comprising BV (2%), candidiasis (13%) , trichomoniasis (8%) and anaerobic vaginitis (9%). Nugent’s scoring delineated study subjects as Grade I (84%), Grade II (14%) and Grade III (2%). Prevalence of various microbes were; *G.vaginalis* (2%), *Candida sp* (16%), anaerobic GNB (12%), anaerobic GPB (9%) and Mycoplasmas (8%). Prevalence of PTB and LBW were; 30(6%) and 59(13%) respectively. Presence of anaerobic GNB and GPB (Nugent’s Grade II organisms) was statistically significant with both PTB (*p*= >0.01, 0.005) and LBW (*p*= 0.001, 0.026).

## Conclusion

Incorporation of a simple diagnostic modality like Nugent’s scoring system in antenatal care can help in early diagnosis and management of asymptomatic LGTIs; thereby reduce associated adverse pregnancy outcomes.

